# Investigating the Correlation Among Chinese EFL Teachers' Self-efficacy, Work Engagement, and Reflection

**DOI:** 10.3389/fpsyg.2021.763234

**Published:** 2021-10-11

**Authors:** Yawen Han, Yongliang Wang

**Affiliations:** ^1^School of Foreign Languages, Southeast University, Nanjing, China; ^2^Center for Second Language Writing Research/School of College English Teaching and Research, Henan University, Kaifeng, China

**Keywords:** Chinese EFL teachers, reflection, teacher education, self-efficacy, work engagement

## Abstract

As the forerunners of education, teachers and their psycho-affective variables have been the focus of numerous studies in the past decades. To add to this line of inquiry, the present study aimed to scrutinize the correlation among English as a foreign language (EFL) teachers' self-efficacy, work engagement, and reflection in the context of China. To do so, three previously validated questionnaires related to each of the variables were distributed among Chinese EFL teachers with various experiences and academic degrees, and a sample of 614 completed the questionnaires. The results of Pearson's Product-moment correlation revealed that the participants' self-efficacy, work engagement, and reflection were positively correlated. Moreover, the results of regression analysis and ANOVA demonstrated that Chinese EFL teachers' self-efficacy and work engagement significantly predicted their reflection. The findings have viable takeaways for EFL teachers and teacher education programs in that they can invest more time and energy in promoting psychological factors in teaching the English language along with pedagogical issues.

## Introduction

Teachers are now universally accepted as the most important part of educational systems (Coombe, 2014, 2020; Farrell, 2014, 2015, 2018; Cheng and Wu, 2016; Nayernia and Babayan, 2019; Derakhshan et al., 2020; Gao and Zhang, 2020; Chu et al., 2021; Coombe et al., 2021). They are the frontline soldiers with academia actions and emotions as their first priority (MacIntyre et al., 2019; Mercer, 2020). This orientation has rooted in post-method pedagogy arguing that teachers are no longer mere consumers of knowledge but theory-makers and active practitioners (Kumaravadivelu, 2001). They are the “pillars” and “architects” of societies (Pishghadam et al., 2021), and they do not enter a class without carrying their own beliefs, values, and emotions (Greenier et al., 2021). Hence, knowing their individual and psychological attributes is of crucial significance in educational milieus. As a result, numerous studies have recently been conducted on teacher-psychology variables (e.g., self-esteem, self-efficacy, motivation, agency, resilience, identity, well-being, burnout, autonomy, etc.). One of the most important teacher-related variables in second/foreign language contexts is self-efficacy that refers to one's self-concept regarding his/her abilities to accomplish a task efficiently (Bandura, 1977; Tschannen-Moran and Woolfolk Hoy, 2001; Fathi and Derakhshan, 2019; Fathi et al., 2021; Liu et al., 2021). First introduced in Social Cognitive Theory (SCT), self-efficacy underscores one's agency, engagement, and control over what he/she does (Bandura, 1997). From this stance, self-efficacy strongly influences the person's reflections, self-organization, goals, and almost all behaviors (Schunk and Meece, 2006).

With respect to teachers, the concept of self-efficacy points to their judgment of their own competence in managing the classroom, engaging students, and performing assigned teaching tasks (Tschannen-Moran and Woolfolk Hoy, 2001). This feeling affects their general orientation toward teaching, their specific classroom behaviors and practices, and even students' academic achievement (Bandura, 1986, 2000; Alibakhshi et al., 2020). Acknowledging this powerful influence, many studies in EFL contexts have explored the association and impact of teachers' self-efficacy on various psychological constructs such as teacher engagement, emotional intelligence, job satisfaction, perfectionism, identity, commitment, burnout among other factors). Furthermore, as a less explored construct related to self-efficacy, teachers' work engagement concerns their job satisfaction, concentration, productivity, positive aspiration, resilience, and adaptability (Greenier et al., 2021). It is a dynamic construct and a state of mind characterized by vigor, dedication, and absorption (Schaufeli et al., 2002). As pinpointed by Faskhodi and Siyyari (2018), work engagement is the positive opponent of burnout. It reflects teachers' quality of professional career and is positively correlated with self-efficacy (Minghui et al., 2018).

Work engagement occurs when an interplay of personal and contextual requirements are fulfilled. In addition to positive inner states, there must be professional attractions available, too. For instance, a positive and friendly organizational culture and structure is a critical factor in getting fully involved in a job. When a teacher is psychologically ready and enthusiastic to teach and external needs are met, he/she does whatever possible to incur outstanding outcomes. One of the offshoots of this deep immersion in work is teacher reflection and reflectivity. Reflection refers to a teachers' careful consideration of his/her instructional practices for the purpose of improving the instruction (Shirazizadeh and Karimpour, 2019; Fathi et al., 2021). It has been considered an integral part of teacher development programs throughout the world as it increases teacher's efficacy, job satisfaction, professional success, emotional intelligence, and interpersonal skills (Braun and Crumpler, 2004).

The coiner of the term *reflection*, Dewey (1933), defines it as “the active, persistent and careful consideration of any belief or supposed form of knowledge in the light of the grounds that support it” (p. 9). Munby and Russell, 1990 explain that reflection starts with a puzzle on which an individual reflects when trying to deal with it. The concept has been influenced by different philosophies, each of which defines reflection from their own perspectives. Akbari et al. (2010), however, proposed a comprehensive model for teacher reflection, based on the extraction from 600 categories and behaviors. The model consisted of six factors, namely, practical, cognitive, affective, met-cognitive, and critical elements, aiming to capture teachers' reflections in dealing with teaching issues. This model of reflection entails a broad range of behavioral aspects and it is interesting to see how it is correlated with other psychological factors affecting teachers. Moreover, research shows teachers' reflections and sense of efficacy are interconnected. They have a two-way association, each can affect the other (Fathi et al., 2021). When a teacher has the confidence of his/her teaching expertise, he/she reflects more on his/her teaching behaviors, plans, and practices and is more eager to pedagogically grow. Although the three constructs of teachers' self-efficacy, reflection, and work engagement share many theoretical underpinnings and function as a nested system, they have rarely been explored simultaneously in EFL contexts especially with a focus on their predictability power in juxtaposition to each other. Against this backdrop, the present study aimed to examine whether self-efficacy and work engagement predict EFL teachers' reflection in the context of China.

## Literature Review

### Theoretical Underpinnings

Different theories underpin the association among teachers' self-efficacy, work engagement, and reflectivity, including positive psychology (PP), self-efficacy theory (SET), work engagement theory (WET), and reflective practice theory (RPT). As a new trend in psychology, PP examines how people can thrive through positive emotions and virtues that make life better (Csikszentmihalyi and Nakamura, 2011; Wang et al., 2021). It is a rebirth of humanistic psychology but puts more emphasis on empirical research (MacIntyre and Mercer, 2014). Moreover, it aims to generate positive emotions, improve engagement, and make life meaningful (Seligman, 2006). Instead of dwelling on negative stressors such as anxiety, tension, and stress, PP gives more weight to positive attributes such as joy, hope, enjoyment, resilience, love, courage, optimism, sense of flow and efficacy, and the like. It builds on three pillars of *positive emotions, positive character traits*, and *positive institutions* (Seligman, 2011). SET, which is a sub-category of social cognitive theory, was proposed by Bandura (1986) and highlights the significance of the individual and his/her perceptions of his/her personal capabilities as two key determinants of successful behavior/outcome. SET calls for a democratic model that stresses that all people are able to be successful, if only they have chances and self-efficacy essential to chase their goals. Nevertheless, the theory does not imply that positive self-efficacy beliefs are the single reason for success and achievement but an interaction of personal, behavioral, and environmental factors are at play.

It is worth noting that the construct of self-efficacy is equal to what PP scholars named “*subjective well-being”* or how people feel about their lives and the quality of their experiences (Diener, 2000). They both try to arouse human strengths including optimism, perseverance, and interpersonal skills (Seligman and Csikszentmihalyi, 2000). As another theoretical basis, WET maintains that an engaged person enjoys a positive attitude represented through a never-ending vitality, energy, and determination to attempt and invest time and effort on a task (Schaufeli et al., 2009). Engagement is a positive state that inspires you to make the most of your talents and desires via as much vigor, dedication, and absorption as possible. In simple words, this theory contends that when an individual feels that the activities at work go perfectly with his/her talents and interests, he/she tries best to generate supreme outcomes. WET also argues that engagement affords extra energy to handle adversities and stressful situations. This highlights the importance of macro-structures as preconditions for the development of PP attributes, especially self-efficacy. The last theory behind this study is RPT proposed by Schön (1983). This theory postulates that deep thinking and reflecting on one's practice during and after the event can improve its quality (Schön, 1983). It also strongly underscores the critical role of intuition in professional practice and hence regards reflection as a practical way of synthesizing implicit knowledge and ability (Kinsella, 2010). It distinguishes between reflection-on-action, which is done after an activity, and reflection-in-action, done during the activity. All these theories signify that the variables of interest in this study coalesce and build on each other.

### The Concept of Self-Efficacy

The notion of self-efficacy was first proposed by Bandura (1977) in his influential social cognitive theory of learning. He used the concept to explain one's skills and expertise in an area of knowledge. Self-efficacy or self-assurance refers to one's belief in his/her own capability in doing a task (Bandura, 2011). This belief influences individual's thoughts, actions, behaviors, motivation, competence, effort, and judgment (Bandura, 1997, 2006). It differs from self-concept, which is a global view of one's ability gained through experience and evaluation, and self-esteem, which refers to one's judgment of his/her self-worth. According to Bandura (2011), self-efficacy beliefs are derived from four different sources, including *enactive mastery experience, vicarious learning experiences, verbal persuasion*, and *physiological arousal*. Enactive experience refers to one's past successful experience of doing a task that can be beneficial for his/her professional development in the future. Vicarious experience is obtained by observing someone else doing a similar task. Verbal persuasion is the appraisal of important people concerning one's ability and performance on a task. Finally, physiological arousal refers to one's emotions and inner states like stress, tension, anxiety, and motivation which can influence his/her efficacy beliefs. It is also worth noting that self-efficacy is task and context-specific in that it may change as the difficulty and domain of a task or activity vary.

### Teachers' Self-Efficacy

The construct of self-efficacy, particularly, permeated into students' and teachers' beliefs about their abilities. As for teachers, self-efficacy has been given different conceptualizations referring to the degree to which teachers believe in their capacity to influence learners' performance; their belief or conviction that they are able to affect how well students learn, or their belief in their own competence to unify and implement series of actions necessary in doing a particular teaching task (Tschannen-Moran and Woolfolk Hoy, 2001). In simple terms, teacher self-efficacy is the ability to make a difference in learners' academic performance (Mok and Moore, 2019). It is comprised of three sub-categories, namely classroom management, student engagement, and instructional strategies. The extensive literature on this construct signifies that teachers with high self-efficacy are better at classroom management and organization and have higher commitment, enthusiasm, satisfaction, motivation, reflection, resilience, and well-being (Skaalvik and Skaalvik, 2014; Zee and Koomen, 2016; Fathi et al., 2020, 2021). Fathi et al. (2020), for example, argue that self-efficacious teachers are more likely to take control of negative emotions and deal with the challenges. If a teacher has a higher perceived ability or self-efficacy, he can influence learners, principals, and even the whole educational context. Thus, it is no wonder that self-efficacy is still one of the most fertile intra-psychic variables in education, especially L2 education.

### Teachers' Work Engagement: Definitions and Dimensions

Work engagement refers to a mental state which is related to work and includes vigor, dedication, and absorption (Schaufeli et al., 2002). Work engagement has received psychologists' attention during the last century following the growing interest in positive psychology (Wang et al., 2021). Accordingly, instead of considering the work associated with negative burdens such as stress and anxiety, positive aspects associated with successful fulfillment of the job are getting more attention (Greenier et al., 2021). Work engagement, in this sense, is considered the opposite of burnout and concerns the ways by which a person devotes more time and energy to doing a task. It is affected by both internal and external factors. Teachers' work engagement is a positive inner state that mirrors their professional life, performance, satisfaction, and quality (Field and Buitendach, 2012). It has three dimensions, namely *vigor* (having energy, resilience, willingness, and persistence when working), *dedication* (a sense of importance, pride, inspiration, enthusiasm, and challenge), and *absorption* (being profoundly engrossed in one's work in a way that he/she enjoys working constantly and time passes quickly in his/her view). It is essential to note that work engagement differs from workaholism in that engagement is a positive trait that produces positive outcomes, while workaholism does more damage than good and produces burnout. Based on the first dimension of work engagement, teachers' intrapersonal variables, especially self-efficacy, may influence the quality, and degree of their involvement in teaching.

### Reflection and Reflective Teaching: Conceptualizations

Reflection is one of the most pivotal elements of teachers' professional development, effectiveness, and well-being (Aleandri and Russo, 2015). It was introduced by Schön (1983) and Dewey (1933) referring to deliberate, systematic, and careful action carried out on the logical reasoning behind an action or idea. A reflective teacher evaluates his/her teaching practices and makes required changes in teaching practices, methods, and assessment to improve learning quality (Hua, 2008; Xu et al., 2015). Teacher reflection is of critical importance in bridging the gap between theory and practice in L2 teacher education which emphasizes providing high-quality teachers (Farrell and Kennedy, 2019). Another conceptualization for reflection was proposed by Schön (1983) using three concepts of *reflection-in-action, reflection-on-action*, and *reflection-for-action* which all aim to improve teaching and learning quality. Reflection-in-action occurs during an instructional practice, reflection-on-action happens after the practice to make remedies, and reflection-for-action is future-oriented and aims to improve or change a future action. As pinpointed by Farrell and Kennedy (2019), teacher reflection can be done through discussions, writing personal journals and diaries, concept mapping, case-based instruction, metaphor analysis, and critical incident analysis. Reflectivity is a dynamic construct and occurs through time and appropriate training. Hence, many educational systems are now diving deeper to figure out the best way to improve teachers' reflection which, in turn, affects their engagement, autonomy, burnout, self-efficacy, perfectionism, and teaching-learning beliefs.

Reflecting on the previous conceptualizations in the literature, Akbari et al. (2010) proposed a model for teacher reflection. Using structural equation modeling, they proposed that teacher reflection can be measured through affective, practical, cognitive, meta-cognitive, and critical categories. The practical element deals with the tools/procedures that teachers use in the practice of reflective teaching. The affective element refers to teachers' reflection on the learners' challenges/learning and the cognitive element includes the attempts done by teachers for professional development, and the meta-cognitive element refers to teachers' own judgment of their practices. Finally, the critical element deals with teachers' perceptions of the socio-political effect of their practices. As the model seems comprehensive, this research takes this model in measuring participants' reflection.

### Empirical Underpinnings

The research on teachers' self-efficacy has witnessed an exponential increase all around the world. It is a pervasive variable in L2 education that draws a demarcation between effective and ineffective teachers. Previous research on this construct indicates that it has a relationship with burnout, optimism, engagement, resilience, reflection, interpersonal communication skills, and well-being (Zee and Koomen, 2016, to name a few; Putwain and von der Embse, 2019; Fathi et al., 2020; Wang and Derakhshan, 2021). Teachers' self-efficacy is dynamic and influenced by personal and social-demographic factors. For instance, Minghui et al. (2018) found that Chinese special education teachers' self-efficacy could be predicted by their social support, salary, and teaching experience. Similarly, in their recent study, Fathi et al. (2021) found a correlation between EFL teachers' self-efficacy and reflection.

Concerning work engagement which is a sense of professional commitment, in the past decades, scholarly interest in the concept has increased. Pertinent studies inspired a shift of attention from teaching burnout to work engagement as a positive psychology variable (Bakker and Albrecht, 2018). However, in L2 education, studies on burnout and work engagement have been under-addressed (Piechurska-Kuciel, 2011). Recently, in a seminal study, Greenier et al. (2021) ran a cross-cultural study on teachers' emotion regulation, psychological well-being, and work engagement. Using a mixed-methods design, they collected data from 108 British and 255 Iranian language teachers. In the end, they found that both emotion regulation and psychological well-being significantly predicted teachers' work engagement. Moreover, in Burić and Macuka (2018) found that work engagement was affected by teachers' positive emotions like self-efficacy. They also argued that teachers with high self-efficacy, had high work engagement, too. Likewise, Minghui et al. (2018) identified that self-efficacy and social support could be predictors of teachers' work engagement. The point that motivated the present research is that most of the available studies on work engagement have been carried out in general education contexts and not in L2 education. Previous works that take the work experience of teachers mainly delve into the negative aspects of it (e.g., Fathi and Derakhshan, 2019). However, Wang et al. (2021) highlight the need for searching positive psychological factors affecting the teachers and learners. Following their call, this research probes into the possible positive correlation between work engagement and teacher reflection.

With regard to teacher reflection, research indicates that it is a vital element of teaching quality, effectiveness, and professional growth (Farrell, 2018). In L2 education, it has been studied in relation to self-efficacy (Yost, 2006), perfectionism (Shirazizadeh and Karimpour, 2019), burnout (Fathi et al., 2021), and emotion regulation (Greenier et al., 2021). It has a positive correlation with self-efficacy but a negative correlation with negative emotions like stress and burnout. The impetus behind running this study was that, although the three constructs of teacher's self-efficacy, work engagement, and reflection have common theoretical bases suggesting that they are not mutually exclusive, the pertinent studies in the literature available are mainly correlational without seeking the possibility of their prediction power in relation to each other. This demerit is also observable in the EFL context of China which equally has separate studies on these variables. Additionally, a simultaneous exploration of these constructs and the predictive power of self-efficacy and work engagement in relation to teacher reflection has been absent in China, to date. Against this shortcoming, the current study was a bid to investigate Chinese EFL teachers' self-efficacy and work engagement as possible predictors of their reflection. More specifically, it sought to answer the following research questions:

1. Is there any statistically significant correlation among Chinese EFL teachers' self-efficacy, work engagement, and reflection?
2. Do Chinese EFL teachers' self-efficacy and work engagement significantly predict their reflection?

## Method

### Participants and Research Context

The target participants of this study were 614 Chinese EFL teachers with different academic qualifications and teaching experiences including both genders (male = 86, female = 528). Their age ranged from 28 to 57. They were currently teaching at different educational levels in China and were selected through convenience sampling and based on their willingness to participate in the study. More demographic information is given in Table 1.

**Table 1:** Participants' profile.

**Background information**	**No**.
**Gender**	
Male	86
Female	528
**Teaching experience**	
1–5	89
6–10	77
11–15	135
16–20	152
21–25	99
26+	62
**Academic degree**	
AA	31
BA	265
MA	213
PhD	35
Other	70
Total	614

### Instruments

#### Questionnaires

##### Teacher Self-Efficacy Questionnaire

To measure Chinese EFL teachers' self-efficacy, the Teachers' Sense of Efficacy Scale (TSES) designed and validated by Tschannen-Moran and Woolfolk Hoy (2001) was utilized in this study. The scale includes 24 items spreading into three sub-components of instructional strategies, classroom management efficacy, and student engagement efficacy. The scale follows a 5-point Likert scale ranging from 1 (nothing) to 5 (a great deal). As for reliability, Fathi and Derakhshan (2019) empirically identified it to be 0.89 in the EFL context of Iran. The reliability of the questionnaire for the present study was also estimated through Cronbach's alpha formula and the alpha turned out 0.87. The reason for choosing this scale was that TSES was the most widely used and approved tool in this domain.

##### Teacher Work Engagement Questionnaire

Engagement was assessed via Schaufeli et al.'s (2002) questionnaire as the most popular existing scale on work engagement. It includes 24 items classified into three underlying dimensions of engagement namely, vigor (9 items), dedication (8 items), and absorption (7 items). The items are scored on a 6-point Likert scale with (0) representing “never” to (6) representing “always”. With regard to the reliability of the scale, the results of Cronbach's alpha revealed a 0.84 coefficient.

##### Teacher Reflection Questionnaire

In this study, EFL teachers' reflection was measured through the English Language Teaching Reflection Inventory developed by Akbari et al. (2010). It encompasses 29 items divided into five dimensions including practical, cognitive, affective, metacognitive, and critical dimensions. The items are based on a 5-point Likert scale with (1) representing “never” and (5) representing “always.” The overall score on all the five sub-scales of the instrument is considered as the degree of EFL teachers' reflection. Concerning the reliability index, the results of Cronbach's alpha indicated a coefficient of 0.91. This instrument was used because it was the result of a thorough analysis of many related tools and covered different dimensions of the construct of teacher reflection.

### Data Collection

To meet the objectives of the study, the data were gathered via both soft and hard copies of three previously validated questionnaires on teachers' self-efficacy, work engagement, and reflection over three months. The respondents were 614 Chinese EFL teachers with different teaching experiences and academic degrees including both genders selected on the basis of convenience sampling technique. The data were gleaned from various language institutes and universities in China. Initially, the researchers provided sufficient clarification of how to fill out the questionnaires for the respondents and assured them that their responses and identity would be kept confidential. Then, the responses of the participants were checked for possible mistakes before being entered into SPSS software for statistical analysis.

### Data Analysis

After entering the collected data into SPSS v. 24, the researchers evaluated the assumptions required for running pertinent statistical techniques. With the preliminary assumptions met, descriptive statistics for each of the three variables were provided together with their correlation that revealed the overall image of the data. To answer the first research question, Pearson's Product-moment correlation was used. Concerning the analysis of the second research question, the researchers took advantage of regression analysis and ANOVA.

## Results

### Preliminary Analyses

The data were analyzed through correlational and regression analyses. There are a number of pre-requisites that legitimize the accuracy of results obtained from these tests. The initial concerns are the normality and lack of outliers. First, by inspecting the boxplots for each variable, 12 cases (Case No. 3, 43, 85, 118, 138, 209. 250, 275, 376, 387, 455, 518) which showed characteristics of outliers were excluded. Then the normality was probed using skewness/kurtosis values. Table 2 reports the descriptive statistics of the obtained results.

**Table 2:** Descriptive statistics.

	**N**	**Min**.	**Max**.	**Mean**	**SD**	**Skewness**	**Kurtosis**
Self-efficacy	602	56.00	120.00	94.5449	13.52075	0.100	−0.652
Reflection	602	52.00	120.00	91.0482	15.68553	0.147	−0.629
Work	602	36.00	119.00	91.6262	19.16566	−0.460	−0.445
engagement							

**Figure 1:**
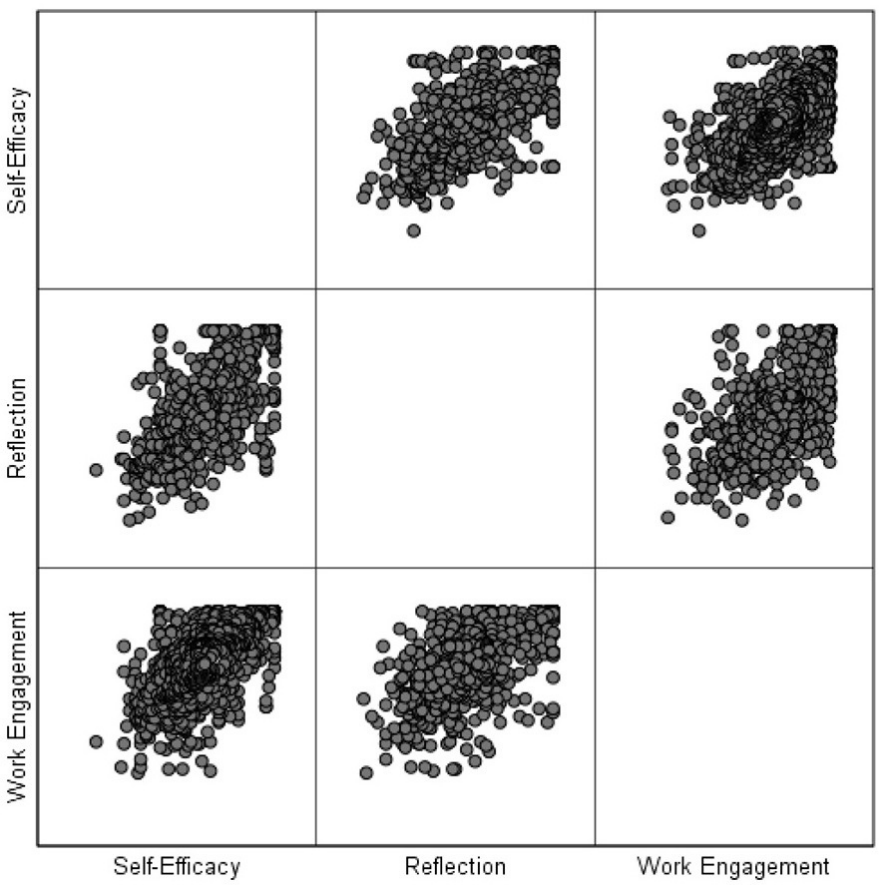
Multiple scatterplot of the relationships between variables.

As displayed in Table 2, the skewness and kurtosis values fell within the range of ±1.96, indicating normal distribution for all data sets (Tabachnick and Fidell, 2013). The next pre-requisite to be checked was the linearity of the relationship between pairs of variables and homoscedasticity. To check these assumptions, a multiple scatterplot was created (Figure 1).

Through inspecting Figure 1, it is evident that the pairs of variables have linear correlations as the spread of dots started from the bottom left and extended to the top right corners in each cell of the matrix (indicating positive correlations). Moreover, no sign of *U*-shaped or curvilinear patterns was observed in the spread of dots, indicating that the linearity of correlations existed between all pairs of variables. Moreover, no funnel-shaped distribution was observed. In other words, the distribution of dots was not narrow at one end and wide at the other end. Therefore, no homoscedasticity problem existed.

### Results

The first research question required running the correlational analyses. After making sure of the normality, the correlations between the predicating and predictor variables were checked through Pearson product-moment correlations (Table 3).

**Table 3:** Correlations among the variables.

		**Self-efficacy**	**Work engagement**	**Reflection**
Self-efficacy	Pearson correlation	1	0.574^**^	0.607^**^
	Sig. (2-tailed)		0.000	0.000
	N	602	602	602
Work	Pearson correlation	0.574^**^	1	0.588^**^
Engagement	Sig. (2-tailed)	0.000		0.000
	N	602	602	602
Reflection	Pearson correlation	0.607^**^	0.588^**^	1
	Sig. (2-tailed)	0.000	0.000	
	N	602	602	602

** *Correlation is significant at the 0.01 level (2-tailed)*.

As reported in Table 3, the correlations among the three variables were significant at the 0.01 level. The correlation between self-efficacy and reflection was 0.607 and the correlation between work engagement and reflection was 0.588. The two predicting variables, i.e., self-efficacy and work engagement had a correlation coefficient of 0.574.

To answer the second research question, a multiple linear regression analysis was performed. As there were significant correlations between each pair of predicting and predicted variables, the first pre-requisite for running the regression analyses was met. Before running the test, the data were checked for multicollinearity, regression normality, and the existence of outliers. Multicollinearity occurs when there is a high correlation between/among the predicting variables. As reported in Table 3, above, the correlation between the two predicting variables was 0.574. Although this suggests a strong correlation, it does not question the divergent validity of the two variables (correlations above 0.7 may be problematic). To make sure that there is no threat of divergent validity, multicollinearity was systematically inspected. The Tolerance value and variance inflation factor (VIF) value are two commonly-used measures when it comes to confirming or rejecting the existence of multicollinearity. For our sample, the VIF value turned out 1.42 and the Tolerence value was 0.71, which shows lack of collinearity. According to Tabachnick and Fidell (2013), the Tolerance value is <0.1 and VIF values above 10 are problematic.

For regression normality, we inspected the normal probability plot. No major deviation was observed from the regression diagonal suggesting the normality. Moreover, a scatterplot of standardized residuals was created to inspect both normality and the existence of outliers. The inspection of the scatterplot showed that the residuals were rectangularly distributed and the majority of the dots were located at the center showing no clear pattern. Moreover, none of the cases in the standardized residual were more than 3.3 or < -3.3, suggesting a lack of outliers. In addition, we inspected the Mahalanobis maximum distance value which was 11.94, which was safely below the critical value of 13.82 for regression models with two predicting variables. Therefore, the non- existence of outliers was also ensured.

Having all the assumptions met, the researchers opted for running a multiple linear regression analysis in order to answer the second research question. The regression model summary showed that *R* and *R*^2^ values were 0.674 and 0.454. The R^2^ value suggests that the regression model explains 45.4% of the variance in the total score of the teacher reflection (Cohen et al., 2003). The difference between R^2^ and adjusted R^2^ (0.454 −0.453 = 0.001) shows that the model has a large generalizability power. Moreover, the Durbin-Watson (DW) index of 1.74 indicated independence errors. Values between 1 and 3 are indicators of independence of errors (Tabachnick and Fidell, 2013).

Table 4 reports the results of ANOVA (*F*_(2, 599)_ = 249.43, *p* = 0.000 < 0.05) for the model, which is considered significant. This means that the model can significantly predict EFL teachers' reflection.

**Table 4:** Regression model: ANOVA.

**Model**		**Sum of squares**	**df**	**Mean square**	**F**	**Sig**.
1	Regression	67,189.956	2	33,594.978	249.430	0.000^b^
	Residual	80,677.647	599	134.687		
	Total	147,867.603	601			

b *Predictors*.

Finally, the regression coefficients, presented in Table 5, indicate the extent to which each predictor variable, i.e., self-efficacy and work engagement, contributes to the prediction of the predicted variable, i.e., reflection. The presented results showed that the largest β value belonged to self-efficacy, (β =0.402, *t* = 10.91, *p* = 0.000 < 0.05), while work engagement had relatively lower value (β =0.357, *t* = 9.7, *p* = 0.000 < 0.05). The inspection of part correlation indices revealed the degree of unique explanation of reflection by each of the predicting variables. Accordingly, self-efficacy explains 10.82 percent (0.329 × 0.329 = 0.1082) and work engagement explains 8.59 percent (0.166 × 0.166 = 0.0859) of the variance in total teacher reflection score.

**Table 5:** Regression model: regression coefficient.

	**Unstandardized coefficients**		**Standardized coefficients**	**t**	**Sig**.	**Part correlation**
	**B**	**Std. Error**	**Beta**			
(Constant)	20.138	3.373		5.970	0	
Self-efficacy	0.466	0.043	0.402	10.913	0	0.329
Work engagement	0.293	0.030	0.357	9.702	0	0.293

**Figure 2:**
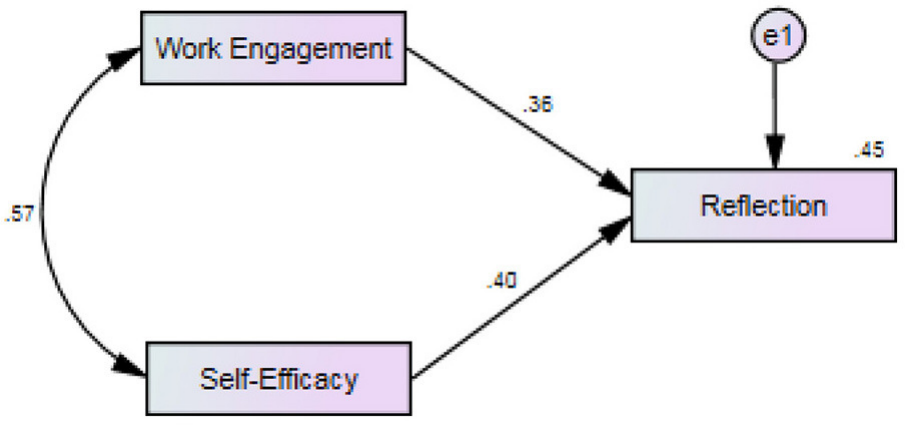
Regression model with standardized estimates.

The final regression model with standardized coefficients is presented in Figure 2.

## Discussion

The present quantitative research study aimed to explore the relationship among self-efficacy, work engagement, and reflection in the EFL context of China. Moreover, it sought to investigate whether teachers' self-efficacy and work engagement significantly predict their reflection or not. Based on the results of Pearson's Product-moment correlation, the correlations among the three variables were significant at the 0.01 level. More specifically, the correlation between self-efficacy and reflection was 0.607 and the correlation between work engagement and reflection was 0.588. Furthermore, the two predicting variables (i.e., self-efficacy and work engagement) had a correlation coefficient of 0.57.4. This finding is in line with those of Burić and Macuka (2018) who ran a similar study in Croatia and identified that self-efficacy has a positive correlation with work engagement. This means that teachers with high self-efficacy will have high work engagement, too. Moreover, the results are consistent with Minghui et al. (2018) who found self-efficacy as a predictor of teachers' work engagement. A possible justification for this finding can be the fact that when EFL teachers are so sure of themselves and their pedagogical expertise to incur learning, they devote more time and energy to their job and passionately involve in the work.

In this study, it was also found that teachers' self-efficacy and reflection are positively correlated. This is on a par with results obtained by Braun and Crumpler (2004) who also found a positive relationship between teachers' reflectivity and efficacy. In light of this, it can be claimed that EFL teachers with high reflection constantly contemplate their teaching practice and how to improve its quality. This makes them enjoy their job, become more efficacious, and believe in themselves.

As a fresh finding, in this study, it was also found that teachers' work engagement is positively correlated with their degree of reflection. It can be argued that these two variables are intertwined in that when a teacher continuously reflects in/on/for his action, he/she gets more engaged in his/her work and more positive outcomes are obtained. The reverse is also true in the sense that a teacher who is highly engaged in his/her work, constantly reflects on his/her own practice so that its quality can be improved. Additionally, the results indicated that Chinese EFL teachers' self-efficacy and work engagement significantly predict their reflection. Although there is a paucity of previous studies on the variables investigated in this research, the results support the claim of Akbari et al. (2010), that reflection is a multidimensional factor and may be related to various psychological and behavioral traits. This is where the present research adds to the body of knowledge in this research domain. To date, the predictive power of self-efficacy and work engagement in relation to teacher reflection has been kept at the margins. A logic behind this prediction might be that Chinese EFL teachers are currently taking giant steps to grow professionally in teaching L2. Hence, their pedagogical competence has considerably improved leading to a high sense of efficacy regarding their abilities to teach. Correspondingly, the amount of energy and time that they invest in teaching English has increased, too. The amalgamation of these improvements in ability, sense of efficacy, and work dedication and engagement made Chinese teachers reflect more on their instructional behaviors and practices.

Recent studies on self-efficacy and work engagement may be of interest here. Fathi et al. (2021) showed that both self-efficacy and reflection are direct and negative predictors of burnout. As mentioned before, work engagement is considered as the positive counterpart of burnout (Faskhodi and Siyyari, 2018). The obtained positive correlations, therefore, were not unexpected and further supports Faskhodi and Siyyari's (2018) claim. The previously unestablished correlation found in this research was the one between reflection and self-efficacy and can be of interest to future research. Moreover, relying on the findings of Greenier et al. (2021), work engagement is considered as positively correlated with emotion regulation. Being a part of emotional regulation, reflection was proved in this research to be correlated by another factor of positive psychology, i.e., work engagement.

Finally, the results showed that self-efficacy was a better predictor of reflection than work engagement. The results are in line with the findings of Fathi et al. (2020) who showed that teacher self-efficacy is a better predictor of another positive psychology factor (Wang et al., 2021), i.e., emotional well-being. They argue that teachers with higher self-efficacy are more likely to improve the whole educational context they are working in; therefore, they are more likely to overcome the challenges. As mentioned before, reflection is theorized by a puzzle, and teacher reflection is defined as teachers' choices in dealing with the challenges. Therefore, the two concepts seem close in theory, and the study brought in practical evidence to support it. All in all, the results of this study indicated that despite many theoretical commonalities among the three constructs examined in this study and the growing correlational investigations in EFL contexts, the current study sparked a light on working on the predictability of these variables which has been largely overlooked in the EFL academic arena.

## Conclusion and Implications

In light of the findings, it can be concluded that Chinese EFL teachers' self-efficacy and work engagement significantly predicted their reflection. It can also be claimed this positive correlation among these variables is attributable to the growing knowledge and competence of Chinese EFL teachers and the supportive and boosting instructional culture in the country. In an educational culture that values and supports EFL teachers, naturally, the pedagogical expertise and self-assurance in transferring knowledge to L2 students increases in teachers. Commensurate with this, they progressively get engaged in their profession and carry out more reflective practices so that the quality of instruction can be constantly enhanced. Therefore, it can be concluded that EFL teachers' self-efficacy, work engagement, and reflection resemble a chain of links that are interconnected and directly affect each other.

The results of this study have practical implications for EFL teachers, teacher trainers, teacher education programs, school principals, policy-makers, and L2 researchers. The results are helpful for pre-service and in-service EFL teachers in that they can raise their awareness of the linkage among their self-efficacy beliefs, work engagement, and reflectivity. Hence, they can dedicate more time and effort to their profession and improve their pedagogical competence by doing reflective practices which multiply their effectiveness and involvement in their job. They can realize that their performance is the outcome of an interplay of many intra and inter-personal variables whose knowledge is a must in EFL contexts. Concerning teacher trainers, the results are beneficial in that they can recognize that teacher training courses should not be confined to practical teaching methods and strategies. Instead, they can improve EFL teachers, especially novice teachers' psychological factors alongside pedagogical direction given in teacher training courses (TTCs). Additionally, the results would be of help to teacher education programs in that they can plan, design, and propose programs in which pre-service and in-service EFL teachers' emotional and psychological variables are considered and appropriate practices are provided. In such programs, teachers should be given strategies to raise their own self-efficacy beliefs, work engagement, and conducting various types of reflective teaching such as journals, diaries, portfolios, seminars, and webinars.

Equally, school principals can benefit from this study in they can establish a democratic, friendly, and equipped classroom environment/climate in which EFL teachers can improve their self-efficacy beliefs, degree of work engagement, and reflectivity. Policy-makers are another group that can use the findings of this study in that they can develop and propose instructional plans, syllabi, and curriculum in which teachers-psychology variables are highlighted and attended. Establishing a general positive classroom culture in academia is a macro-plan that is beyond EFL teachers' responsibility. Finally, L2 researchers can take advantage of this study by running similar studies in other contexts, focusing on other teacher-related variables, and use other research instruments. They can also benefit from longitudinal and cross-cultural studies on teachers' self-efficacy, work engagement, and reflection to see if the dimensions and components of these variables differ across cultures or not.

### Limitations and Suggestions for Further Research

Despite its strengths and novelty in exploring three critical teacher-related variables simultaneously using a large sample size, the present study, like any research, suffered from some limitations. First, the first limitation is that the data were gathered only from China which has special socio-cultural features that may prevent the generalizability of results to other contexts. Second, like many other psychological variables, measuring EFL teachers' self-efficacy, work engagement, and reflection cannot be accurate using only a questionnaire. Thirdly, teachers' professional background, demographic factors, socio-economic status, lifestyle, and ethnicity would influence psychological factors which were not controlled in this study. To compensate for these limitations, future studies can be done by L2 researchers on the same variables as this study but using a mixed-methods research design. Further empirical studies are also needed on work engagement which is a fresh topic in SLA. Moreover, as most of the psychological variables are dynamic and change over time, future studies are recommended using longitudinal designs to unpack the developmental trajectories of these variables. Finally, avid researchers can use qualitative tools such as interviews, reflective journals, and audio diaries to gather data on teachers' intra-psychic factors and provide a more comprehensive image of their vacillation and development.

Further studies may also be interested in cross-cultural studies (e.g., Greenier et al., 2021) to delve into how these psycho-emotional variables can be related, and how their intricate relationships can pave the way for future research. Such studies may greatly contribute to extending our understanding of the less-frequently explored factors, like work engagement and reflection, in different contexts. They may also take into account interpersonal factors such as credibility, rapport, confirmation, immediacy, stroke, etc. (Xie and Derakhshan, 2021; e.g., Pishghadam et al., 2021) and explore the mediating effect of such factors. Moreover, as discussed above, the three factors explored in this study are parts of positive psychology factors (Wang et al., 2021). Future studies may be interested in finding the association of these variables with other factors of positive psychology. Finally, the dynamicity of psychological factors should not be neglected (Kruk, 2021; Wang and Derakhshan, 2021). More studies are required in different contexts to reach a comprehensive and more accurate understanding of the associations of the variables explored in this study.

## Data Availability Statement

The original contributions presented in the study are included in the article/supplementary material, further inquiries can be directed to the corresponding authors.

## Ethics Statement

The studies involving human participants were reviewed and approved by Southeast University and Henan University Academic and Ethics Divisions. The patients/participants provided their written informed consent to participate in this study.

## Author Contributions

YH: conceptualization, funding, data collection, and writing of introduction and literature review sections. YW: data collection, data analysis, and writing of research methodology, discussion as well as conclusion sections. Both authors listed have made a substantial, direct and intellectual contribution to this original research, and approved it for publication.

## Funding

This study was granted by Philosophy and Social Sciences Foundation of China, entitled Research on the Language Education Policy and Planning for International Students from Countries along the Belt and Road, (Grant No. 19BYY037); and also sponsored by a Teacher Education Research Project: A Study on Chinese EFL Teachers' Emotion Regulation, Resilience and Their Work Engagement Based on Positive Psychology (Grant No. YB-JFZX-22) of Henan University, China.

## Conflict of Interest

The authors declare that the research was conducted in the absence of any commercial or financial relationships that could be construed as a potential conflict of interest.

## Publisher's Note

All claims expressed in this article are solely those of the authors and do not necessarily represent those of their affiliated organizations, or those of the publisher, the editors and the reviewers. Any product that may be evaluated in this article, or claim that may be made by its manufacturer, is not guaranteed or endorsed by the publisher.
